# Physical rehabilitation program for cardiorespiratory health and quality of life among breast cancer survivors in UAE: protocol for a randomized control trial

**DOI:** 10.1186/s13063-024-08192-9

**Published:** 2024-06-04

**Authors:** Fatima Abdul Rashid, Wajiha Anwar, Praveen Kumar Kandakurti, Animesh Hazari

**Affiliations:** College of Health Sciences, Gulf Medical University, Ajman, United Arab Emirates

**Keywords:** Breast cancer survivor, Physical rehabilitation, Cardiorespiratory health, Quality of life

## Abstract

**Background:**

Cancer is a medical condition where some cells of the body reproduce uncontrollably and metastasize to other parts of the body. The burden of the disease is significantly high both at the global and national levels. In UAE, cancer was found to be the third leading cause of death. Breast cancer has been ranked first due to its prevalence, incidence, and mortality in UAE. Breast cancer survivors have significantly poor cardiovascular tolerance which affects their quality of life (QoL), even after the carcinoma has been treated or removed. Thus, the protocol aims to analyze the changes in cardiovascular endurance and QoL domains for breast cancer survivors in the United Arab Emirates using a long-term 2-month physical rehabilitation.

**Methods:**

A total of 60 breast cancer survivors would be included in the study using a randomized controlled allocation of a 2-month physical rehabilitation intervention program with 3 months of follow-up. The intervention would target the cardiovascular endurance component of the participants to improve their physical well-being and quality of life ultimately.

**Discussion:**

The findings of the study would have high clinical significance among breast cancer survivors in the UAE. The proposed physical rehabilitation program could be beneficial in improving cardiovascular endurance and thereby reduce the risk of mortality among breast cancer survivors. In addition, the physiological benefits of the exercise program could improve their quality-of-life domains including physical, mental, and social well-being. On a larger view, it could also help to reduce the economic burden on the health system due to associated complications.

**Trial registration:**

ClinicalTrials.gov NCT06013527. Registered on 28 August 2023.

## Introduction

### Background and Rational {6a}

Cancer is a medical condition where some cells of the body reproduce uncontrollably and metastasize to other parts of the body [1]. The burden of the disease is significantly high both at the global and national levels. In the year 2020, 19.3 million total new cases of cancer were recorded, and cancer deaths were estimated to be 10.0 million, for both males and females. Out of this, Asia accounts for 58.3% of cancer deaths, while Europe constitutes about a quarter of total cancer deaths, estimated at 19.7%, and America has 14.2% cancer-related mortality [2]. In 2022, 2.3 million women were diagnosed with breast cancer, hence being the most common cancer in women in 157 countries out of 185 [2]. In UAE, cancer was found to be the third leading cause of death where breast cancer accounts for 21.4% [3]. Therefore, breast cancer was ranked first due to its prevalence, incidence, and mortality [3, 4]. The high burden of the disease prompts the medical community to explore and advance treatment options. This effort is seen in the decrease in the overall mortality rate by 40% from 1998 to 2012 [5]. However, breast cancer survivors have significantly poor cardiovascular tolerance which affects their quality of life (QoL), even after the carcinoma has been treated or removed.

Poor cardiovascular endurance has been very commonly reported among breast cancer survivors [6]. The cardiorespiratory system executes the main function of our circulatory system and provides energy for body movements. Poor functioning and endurance directly affect the individuals’ level of independence and ability to participate in the physical, mental, and social environment affecting the overall QoL. Furthermore, it is well known that QoL domains are interrelated where physical rehabilitation is of utmost importance as it can help to address all domains. In addition, physical rehabilitation forms an important part of breast cancer management pre- and post-surgical. In the pre-surgical phase, rehabilitation can help the individual to sustain the ill effect of post-surgical complications better. However, it is the post-surgical phase that requires more attention and rehabilitation strategies to combat the situation and help the individual regain their functional level near normal.

The importance and benefits of physical rehabilitation among breast cancer survivors have been well reported with evidence in the literature [7]. Studies report that physical rehabilitation techniques and exercise regimens can help to reduce pain, fatigue, weakness, and lymphedema and thus improve QoL [8]. Integrated structured exercise programs have been defined and prescribed to check their benefits over the QoL domains [9–11]. In addition, a study reported that while structured exercise programs should focus on improving cardiorespiratory fitness and muscular strength during exercise training, these programs should consider physical activity outside of training, if well-tolerated, to potentially further lower fatigue and improve QoL in cancer survivors [12]. Thus, an individual-centric approach could be more beneficial to match individual needs efficiently.

Although a recent study reported a higher incidence and prevalence of breast cancer in the UAE population [4], no published data was found on the application of evidence-based exercise regimens, rehabilitation programs, and guidelines for breast cancer survivors in the UAE. Therefore, the proposed research project aims to improve cardiovascular endurance in the QoL domains among breast cancer survivors in the United Arab Emirates using physical rehabilitation and recommended exercise programs.

### Objective {7}

The objective is to analyze the changes in cardiovascular endurance and QoL domains for breast cancer survivors in the United Arab Emirates using a structured physical rehabilitation program for 2 months with a follow-up after 3 months.

### Trial design {8}

Prospective, parallel-group, controlled, equal allocation ratio, randomized controlled trial of breast cancer survivors (hospitalized or homecare).

## Methods

### Study setting {9}

Patients will be recruited for the study primarily at Thumbay Physical Therapy and Rehabilitation Hospital and Gulf Medical University, Ajman, UAE. Other hospitals and organizations catering to breast cancer support to the survivors will also be explored in all seven emirates of UAE. The proposed list of hospitals for approval is as follows:

- Burjeel Hospitals, Dubai
- American Hospital, Dubai
- Emirates Cancer Society, Ajman
- Al Tawam Hospital, Abu Dhabi
- Czech Rehabilitation Hospital, Dubai
- Burjeel Royal Hospital, Abu Dhabi
- VPS Healthcare Cancer Center, Abu Dhabi
- Al Mafrah Hospital, Abu Dhabi
- Sharjah University Hospital, Sharjah
- Gulf International Cancer Centre, Abu Dhabi
- Zuleikha Hospital, Sharjah
- Mediclinic City Hospital, Dubai
- Cleveland Clinic, Abu Dhabi
- Friends of Cancer, Sharjah

### Eligibility criteria {10}

During the screening and study period, the investigators will assess the patients for eligibility and collect demographic and medical history. The inclusion criteria are as follows:

- Breast cancer survivors who have undergone chemotherapy, mastectomy, and cleared for post-surgical physical rehabilitation.
- Age group 18 years to 65 years
- Female participants (breast cancer survivors)
- Diagnosed with stage I to stage IIIa breast cancer. (As other stages of cancer include metastasis)

The exclusion criteria are as follows:

- Patients diagnosed with prior cardiorespiratory disorders, cognitive dysfunction,
- and other health problems that would prevent safe participation
- Active breast cancer patients
- Participants who engaged in physical activity like the intervention group in any form

### Additional consent provisions for collection and use of participant data and biological specimens {26b}

Not applicable; the study does not use any biological specimens or provisions for additional data.

### Explanation for the choice of comparators {6b}

The comparators used in the study were chosen based on the study design and research question and to match the objectives of the study.

### Interventions {11a}

The intervention group will receive a structured exercise adapted from the BReast cancer and EXercise (BREX) program which has shown significant improvement in Quality of Life and Physical Fitness among breast cancer survivors [15]. The exercise program will continue for 2 months, and a follow-up will be done after 3 months. The exercise program is detailed in Table 1. Exercise would be prescribed and supervised by the physical therapist (FR and WA). The control group will continue with their routine life and will receive standard care given by the hospital.

**Table 1:** Description of the proposed physical rehabilitation program among breast cancer survivors

Components of physical rehabilitation program Supervised—2 times a week Individually tailored program—3 times a week	Frequency (5 times a week for 2 months)
Supervised Training: Two types over alternate weeks: 1) 60-min step aerobics class 2) 60-min circuit training class Each class will include warm-up and cool-down periods both lasting 10–15 min. The maximum target rate of perceived exertion (RPE) would be set at 14–16 on a 20-point Borg scale, a level of exercise that feels “somewhat hard” or “hard” and coincides with about 86–92% of maximal heart rate, 76–85% of maximal VO_2_, and 5–7 metabolic equivalents (METs) ideal for improving the related domains of QoL and cardiovascular endurance [16]. During the first 3 weeks, the participants would exercise at a lower level of RPE (approximately 11–13), to adapt to the training	Twice a week
Unsupervised home program: Home training would consist of endurance training such as walking, Nordic walking, or aerobic training, but it would also include jumps and leaps like step aerobics (96 jumps and leaps per home training session) to promote bone health [15]. Warm-up and cool-down exercises, such as marching or climbing upstairs would be recommended before and after the home training session. Home training would be aimed so that the total training would comprise a minimum of five training sessions per week	Three times a week

### Strategy for Treatment modification and adherence {11b}{11c}

Participants will be removed if there is a change in the medical condition, if there are adverse effects noted, or if they choose to drop out. As the intervention is individually tailored, the required modification will be made depending on the patient’s physical ability to adapt to the given exercise protocol. The home program would be randomly and frequently monitored over audio or video phone calls along with a daily logbook which would be maintained and entered by the therapist after discussion with the patient.

### Concomitant care {11d}

No participant will be restricted from receiving external care, if any participant in the intervention or control group is matching with the exercise program of the intervention group, the data would be excluded for statistical analysis.

### Outcomes {12}

- Cardiorespiratory Health
- Quality of life

### Participant timeline {13}

**Fig. 1:**
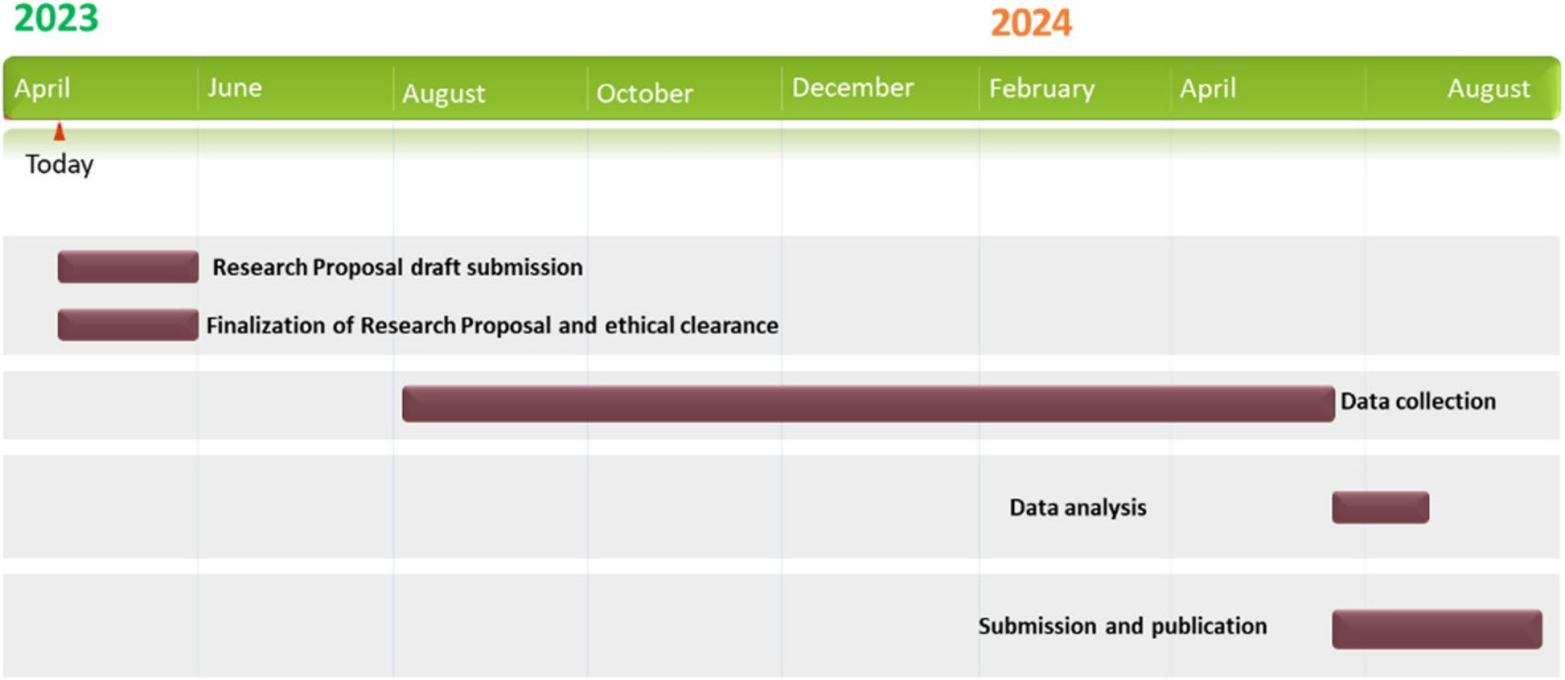
Timeline for the proposed study

The schedule and plan of the study are illustrated in Fig. 1.

### Sample size {14}

The sample size was calculated using the formula for comparison of two means. The outcome variable used was VO_2_ max (cardiorespiratory endurance variable) which was taken from a previous study [13]. The details on sample size calculation are as follows for 95% confidence interval and power set at 80%.

$$\begin{array}{c}N=\frac{2(Z{(1-\alpha/2)+\mathrm{Z\beta})}^2\times\mathrm\sigma^2}{\mathrm d^2}\\N=\frac{2{(1.96+0.84)}^2\times5.9^2}{3^2}\\N=\frac{545.82}9\\N=60.6\end{array}$$

Considering the 20% dropout and loss to follow-up, the total sample size was 72 (36 in each group).

### Enrollment, intervention, and recruitment plan {15}

**Fig. 2:**
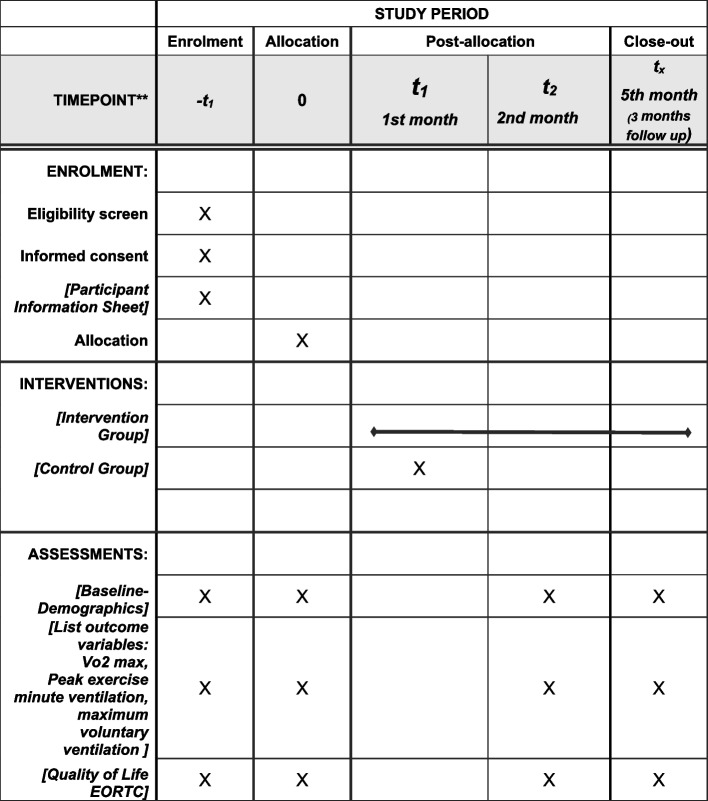
Schedule of enrolment and interventions for the protocol

The participants in the study would be recruited under the purposive sampling method. In addition, hospitals from all emirates would be contacted for sharing the contacts of potential participants from the medical records to meet the target sample size. The study follows the Standard Protocol Items Recommendations for Interventional Trials (SPIRIT) guidelines (Fig. 2) [14]. The recruitment of participants will be illustrated using the CONSORT flow diagram [15].

### Allocation of participants and intervention: randomization and blinding {16a} {16b} {16c} {17a} {17b}

Participants would be allocated to two groups, randomized into equal allocation ratio (1:1) using a simple randomization method through a sealed envelope, with 36 randomized to intervention and 36 to control. Randomization for subject allocation would be double-blinded (conducted by a third party not involved in research) and facilitated through a person not involved in the study. Allocation of intervention will not be blinded and conducted by two researchers in the study FR and WA (physical therapists).

### Data collection {18a}

Data will be collected using the data collection sheet which would focus on the demographics and primary outcome variables using reliable and valid tools. Cardiorespiratory health will be evaluated by analysis of the submaximal VO_2_, peak exercise minute ventilation, and direct maximal voluntary ventilation. The 2-km walk test (UKK walk test, Tampere, Finland) would be done along to calculate the submaximal VO_2_ max and spirometry will be done to record the ventilation values measured using Cosmed Fitmate Pro [16–18]. The QoL will be assessed with the European Organization for Research and Treatment of Cancer Quality of Life Questionnaire (EORTC QLQ-C30) (version 3) [19].

### Follow-up {18b}

All participants in the intervention group would be followed up after 3 months of training sessions. The demographics and primary outcome would be reassessed to analyze the effects on the baseline, at the end of intervention after 2 months and follow-up at 5 months. Any adverse effects at the time of follow-up would also be recorded.

#### Data management: storage and confidentiality {19} {27}

The raw data would be systematically synthesized into an Excel sheet and stored in the institutional data repository system for further analysis for 3 years. The data would be managed both in soft and hard copies. Any personal information of the participants relating to direct identity would not be used for any purpose, and data confidentiality would be ensured by the investigators.

### Plans for collection, laboratory evaluation, and storage of biological specimens for genetic or molecular analysis in this trial/future use {33}

Not applicable. The study does not include the collection, evaluation, and storage of laboratory data through biological specimens for future use.

#### Data analysis {20a} {20b} {20c} {21b}

The data would be analyzed using the SPSS software package version 29. Descriptive and inferential statistics will be done to test the desired hypothesis. Paired and unpaired *t*-tests would be conducted to analyze the changes in the primary outcomes for within and between group analysis. Repeated measures of ANOVA would be done to determine the changes at baseline, end of intervention, and follow-up for the intervention group. If the trial needs to be terminated due to any reasons, interim analysis would be done for the average period of the trial for all participants and the trial would be terminated on a decision from the senior researchers (AH and PK). No adjusted analysis is planned for this research. Missing data would be analyzed through intention-to-treat analysis.

### Plans to give access to the full protocol, participant-level data, and statistical code {31c}

The protocol would be published and dataset would be provided in the repository for public access after the completion of the study.

### Oversight and data monitoring {5d} {21a}

The data would be monitored monthly by the institutional research committee comprised of senior researchers from the study (AH and PK) along with the head of the department and head of the institution. Any administrative and organizational support would be provided by the College of Health Sciences, Gulf Medical University, United Arab Emirates.

### Adverse event reporting and post-trial care {22} {30}

If any adverse effects would be noted and immediately reported by the supervising investigators with record maintenance. If required, participants would be given appropriate treatment and post-trial against any harms at the Thumbay Hospital, UAE.

### Frequency and plans for auditing trial conduct {23}

Not applicable.

### Protocol amendment reporting {25}

Any essential changes in the protocol would be intimated to the ethical committee and consequent changes would be made in the ethical approval letter along with the participant information sheet.

### Dissemination plans {31a}

The findings of the study would be published in quality, peer-reviewed, high-impact factor-indexed journals. Also, findings would be presented at the national and international conferences related to breast cancer.

## Discussion

The findings of the study would have high clinical significance among breast cancer survivors in the UAE. The proposed physical rehabilitation program could be beneficial in improving cardiovascular endurance and thereby reduce the risk of mortality among breast cancer survivors. In addition, the physiological benefits of the exercise program could improve their quality-of-life domains including physical, mental, and social well-being. On a larger view, it could also help to reduce the economic burden on the health system by providing solutions to long-term complications. In the present situation, cancer rehabilitation is one the most important focused themes addressed by the UAE and the findings of this study would be useful for the government policies and planning of long-term management of breast cancer survivors. Clinically, physical therapists could use the findings in making clinical decisions to address the concerns of this population. We assume that the rehabilitation protocol used in the proposed study will be clinically significant to enhance the cardiorespiratory health and quality of life among breast cancer survivors. This would enhance their overall well-being and give better opportunities to rebuild their life.

### Trial status

Protocol version 1, following suggestions from the internal and external review committees. The final protocol draft with ethical approval was obtained in May 2023. The data recruitment will begin in September 2023.

## Data Availability

No datasets were generated for this protocol. The corresponding author and Institutional Research Committee of the department will have access to the final trial dataset. Datasets generated or analyzed in this protocol study will be provided by the corresponding authors upon reasonable request.
